# CRISPR-Guided Proximity Labeling of RNA–Protein Interactions

**DOI:** 10.3390/genes13091549

**Published:** 2022-08-27

**Authors:** Mingxing Lu, Zuowei Wang, Yixiu Wang, Bingbing Ren

**Affiliations:** 1 Department of Oncology, Fudan University Shanghai Cancer Center, Shanghai Medical College, Fudan University, Shanghai 200032, China; 2 Institute of Synthetic Biology, Shenzhen Institutes of Advanced Technology, Chinese Academy of Sciences, Shenzhen 518055, China; 3 Department of Hepatic Surgery, Fudan University Shanghai Cancer Center, Shanghai Medical College, Fudan University, Shanghai 200032, China; 4 Department of Pulmonary and Critical Care Medicine, Regional Medical Center for National Institute of Respiratory Disease, Sir Run Run Shaw Hospital, School of Medicine, Zhejiang University, Hangzhou 310016, China; 5 Cancer Center, Zhejiang University, Hangzhou 310058, China; 6 Sir Run Run Shaw Hospital, School of Medicine, Zhejiang University, Hangzhou 310016, China

**Keywords:** CRISPR-Cas13, bioID/APEX, proximity labeling, RNA elements, RNA–protein interaction

## Abstract

Proximity labeling employs modified biotin ligases or peroxidases that produce reactive radicals to covalently label proximate proteins with biotin in living cells. The resulting biotinylated proteins can then be isolated and identified. A combination of programmable DNA targeting and proximity labeling that maps proteomic landscape at DNA elements with dCas9-APEX2 has been established in living cells. However, defining interactome at RNA elements has lagged behind. In combination with RNA-targeting CRISPR-Cas13, proximity labeling can also be used to identify proteins that interact with specific RNA elements in living cells. From this viewpoint, we briefly summarize the latest advances in CRISPR-guided proximity labeling in studying RNA–protein interactions, and we propose applying the most recent engineered proximity-labeling enzymes to study RNA-centric interactions in the future.

## 1. Introduction

**Figure 1:**
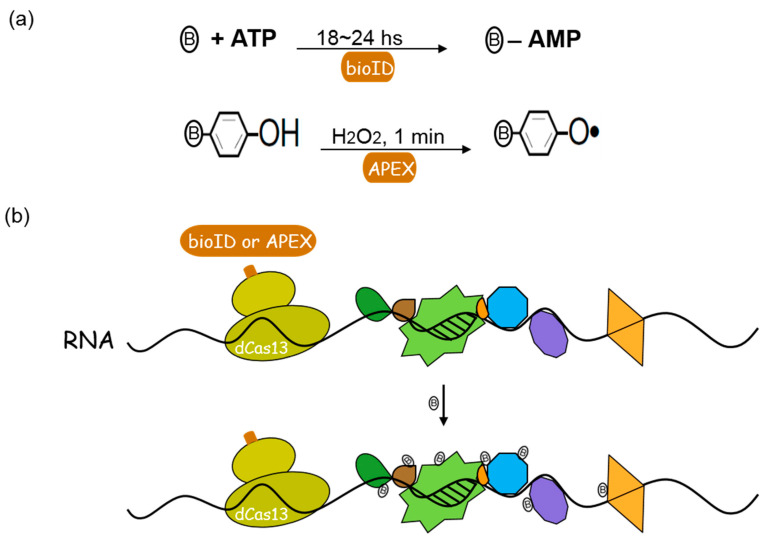
Schematic representation of CRISPR-dCas13-guided proximity labeling. (**a**) Proximity labeling reaction with bioID or APEX. BioID utilizes the supplied free biotin and endogenous ATP to convert biotin to bioAMP radicals, which react with lysine residues on proximate proteins. This reaction usually requires 18~24 h to obtain enough biotinylation materials for quantitative proteomics. Peroxidase-based APEX or APEX2 oxidizes biotin-phenol into massive short-lived biotin-phenoxyl radicals that react with proximate proteins in electron-rich amino acid side chains under the addition of hydrogen peroxide. (**b**) RNA-targeting CRISPR-dCas13-mediated proximity labeling. Catalytically inactivated CRISPR-Cas13 (dCas13) fused with proximity labeling enzyme bioID or APEX, dCas13 fusion protein was directed by CRISPR to target RNA of interest and biotinylate proteins proximal to the RNA in living cells.

RNA–protein interactions are pervasive in regulatory RNAs and RNA-mediated processes in cells [1,2,3]. A growing number of diseases are now thought to be causally associated with aberrant RNA–protein interactions [4,5,6]. Consequently, there is a great demand for methods that enable the rapid identification of interacting partners of specific RNAs. Previously, we reported a proximity-dependent protein labeling method to detect RNA–protein interactions (RBPL) in living cells [7,8], and another method, RaPID, was reported by another group [9,10]. In these studies, the RNA scaffolding motif was used to mediate the recruitment of bioID or its derivative, BASU, demonstrating promising biotinylation around specific RNAs. The drawbacks to these methods, however, are the RNA scaffolding motifs we used to recruit bioID needed to be incorporated into target RNA sequences. Another limitation is the need to express the transgenic RNA scaffolding motifs that may not properly fold or function in cellular physiology. To address these issues, one might use RNA-targeting CRISPR-Cas13 systems to guide bioID or APEX2 to probe endogenously expressed RNAs in a native context (Figure 1a).

## 2. CRISPR-Cas13-Mediated Proximity Labeling for RNA–Protein Interaction Detection

Recently, researchers have applied the RNA-targeting CRISPR-Cas13 system to proximity labeling to achieve programmable RNA-interacting proteins biotinylation [11,12,13,14] (Figure 1b). In these studies, catalytically deactivated Cas13 (dCas13) was fused with proximity-labeling enzyme bioID (BASU or TurboID) or APEX (APEX2) to target RNA of interest and tag its associated proteins [11,12]. BioID is developed from a promiscuous biotin ligase birA* mutant for proximity labeling. BASU is a newly derived variant of promiscuous biotin ligase mutant which uses biotin as a substrate to generate radicals [9]. These radicals can diffuse to neighboring milieu and react with proximal proteins, and BASU’s labeling radius is approximately 10 nm. BASU was fused with dCas13 to detect interacting proteins of specific lncRNAs in native cellular context [11]. TurboID is another version of biotin ligase mutant. TurboID, or miniTurbo, which was developed by yeast display-based directed evolution by Branon et al., has been mutated to generate reactive biotin much faster [15]. However, APEX2 is more efficient than TurboID. APEX2, an engineered ascorbate peroxidase, represents another class of proximity-labeling enzymes. APEX2 catalyzes biotin-phenols into reactive biotin-phenoxyl radicals that diffuse to the neighboring milieu and react with proximal proteins in electron-rich amino acid side chains. Its labeling radius is estimated to fall within 200~300 nm [16]. dCas13 was fused with APEX2 to target specific cellular RNAs for RNA-centric interactome dissection in living cells [13,14]. Hence, CRISPR-guided proximity labeling has been established and demonstrated substantial target labeling in a native cellular context. 

Taken together, these new methods expedite the analysis of RNA interactome in living cells. Compared with previous RBPL or RaPID labeling, CRISPR-guided proximity labeling method is programmable and easy-to-use, which enables arbitrary RNA targeting and the biotinylation of RNA-associated proteins. Moreover, researchers can build inducible CRISPR-guided proximity labeling enzyme fusion cell lines, allowing the spatiotemporal control of proximity labeling activity and the mitigation of high background of biotinylation. CRISPR-guided proximity labeling methods are usually validated with well-known RNA–protein interactions first and then the unknown RNA interactome was explored with mass spectrometry to expedite the analysis of endogenous RNA–protein interactions. Similarly, Zhang et al. reported a new proximity labeling system by using PUT-IT to label proximate proteins of specific RNAs [17]. However, the ligase substrate bio-PupE is a relatively large molecule and cannot diffuse across membranes inside the cell [18].

Although bioID and APEX2 are useful tools for proximity labeling, they have some common drawbacks. For example, bioID takes 18~24 h of reaction time to obtain enough labeled materials for quantitative proteomics [19,20]. Such slow kinetics is not suitable for bioID to probe dynamic processes. TurboID is much more active than bioID and is able to produce robust biotinylation around 10 min of incubation [21]. However, turboID has some inherent issues, such as protein instability, non-specific biotinylation and cell toxicity under certain conditions. Experiments using APEX2 have strong promiscuous labeling within 1 min, which is predominantly used for subcellular compartmental proteomics. Since the reported methods still have some limitations in their current form, a proximity-labeling enzyme with higher efficiency is therefore strongly desired.

## 3. New Engineered Enzymes for Proximity Labeling

Recently, Kohki et al. reported AirID, an *E. coli* birA-derived enzyme for protein–protein interaction detection [22]. In this work, AirID was used to identify proteins that interact with the target protein efficiently and had less cell toxicity over time. These results suggest that AirID is suitable for proximity biotinylation. Although AirID is much faster than bioID, it still requires hours of labeling time. Zhao et al. reported a small and robust enzyme, ultraID [23]. UltraID was fused to argonaute2 and successfully identified known argonaute2-associated proteins. Additionally, the membrane-associated interactome of coatomer, a coat protein complex of COPI vesicles [24], was defined with UltraID at a high temporal resolution. The engineered ultraID enzyme exhibited efficient labeling activity similar to TurboID but with less background of biotinylation. Thus, we anticipate that ultraID might have excellent performance to label RNA proximate proteins when fused to CRISPR-dCas13.

Although numbers of methods have been developed to probe RNA activity, improving RNA-centric targeting affinity and sensitivity is still in early development. It is also expected that a programmable RNA-targeting system could broadly target cellular low-abundance transcripts. The combination of evolved proximity-labeling enzymes and RNA-targeting technology would further expedite the functional analysis of endogenously expressed RNAs in the future.

## 4. Discussion and Perspective

The proximity labeling method employs promiscuous biotin ligases or peroxidases to label proximate proteins. This method is most commonly used to probe protein–protein interactions and the constituents of subcellular compartments refractory to biochemical isolation [16,25]. However, RNA–protein interactions detection by proximity labeling expands the applicability of this method. CRISPR-guided RNA proximity labeling has the potential to reveal spatial features of RNA-interacting macromolecules and dissect disease-associated RNAs. This RNA-centric method could be a generalizable strategy for studying complex interactions of host cellular factors with RNA viruses. For example, the evolved infection and replication in host cells are essential for RNA virus survival and transmission. Co-opting the interactions of the viral RNA genome and host factors is a shared replication strategy of the RNA virus [26]. Potentially, developing methods of CRISPR-guided RNA proximity labeling could be used to target RNA virus genome in infected cells to identify viral RNA-interacting host factors and to dissect molecular mechanism of RNA virus pathogenicity.

Proximity labeling is particularly suitable for probing insoluble or inaccessible subcellular molecules and for detecting weak or transient intermolecular interactions [16,22,23,25]. To date, APEX2 and bioID have been successfully applied to study a variety of proteins and processes in living cells. In combination with RNA-targeting CRISPR-dCas13, proximity labeling can further expand the toolbox for RNA-centric analysis. However, the CRISPR-guided RNA proximity labeling method discussed here has some limitations in its current form. For example, this method is unable to distinguish between directly protein-binding or indirectly proteins associated with RNA. Moreover, the target sequence in CRISPR-dCas13 needs to be considered, such as the folding of RNA and its accessibility or not in living cells. This also requires optimization to adapt to specific cellular contexts. We anticipate that the combination of the CRISPR-dCas13 system and newly engineered proximity-labeling enzymes will further enhance our ability to interrogate RNA–protein interactions.

## Data Availability

Not applicable.
